# Sputum AFB in tuberculous pleural effusion

**DOI:** 10.4103/0970-2113.80345

**Published:** 2011

**Authors:** Viroj Wiwanitkit

**Affiliations:** *Wiwanitkit House, Bangkhae, Bangkok 10160, Thailand 10160. E-mail: wviroj@yahoo.com*

Sir,

I read the recent publication sputum on AFB in tuberculous pleural effusion by Chaudhuri *et al*.[1] with great interest. Chaudhuri *et al*. concluded, “Careful and thorough sputum examination in cases of tuberculous pleural effusion may help as a diagnostic tool and it has therapeutic and epidemiological implications”.[1] I accept that the sputum AFB might be an alternative choice, especially for those settings with limited resource, due to low cost. However, there are many concerns. Very low sensitivity of the test is not high and this can result in underdiagnosis.[2] The use of other advanced diagnostic test is required. Recently, Kumar *et al*. proposed that a combination of pleural fluid with sputum sample and N-PCR improved the diagnosis of pleural tuberculosis.[2]

## References

[1] Chaudhuri AD, Bhuniya S, Pandit S, Dey A, Mukherjee S, Bhanja P (2011). Role of sputum examination for acid fast bacilli in tuberculous pleural effusion. Lung India.

[2] Kumar P, Sen MK, Chauhan DS, Katoch VM, Singh S, Prasad HK (2010). Assessment of the N-PCR assay in diagnosis of pleural tuberculosis: Detection of M.tuberculosis in pleural fluid and sputum collected in tandem. PLoS One.

